# Synthesis of activated carbon from sewage sludge using phosphoric acid as activating agent

**DOI:** 10.1007/s11356-025-36516-y

**Published:** 2025-05-23

**Authors:** Gara Carreño Hernández, Elisenda Pulido Melián, Dunia E. Santiago

**Affiliations:** 1 Dpto. de Ingeniería de Procesos, Universidad de Las Palmas De Gran Canaria, Campus Universitario de Tafira, 35017 Las Palmas, Spain; 2 Instituto de Estudios Ambientales y Recursos Naturales (I-UNAT), Grupo de Fotocatálisis y Espectroscopía Para Aplicaciones Medioambientales (FEAM), Edificio Polivalente 1 del Parque Científico Tecnológico de La Universidad de Las Palmas de Gran Canaria, 35017 Las Palmas, Spain

**Keywords:** Sewage sludge, Activated carbon, Magnetic activated carbon, Methylene blue, Phosphoric acid, Regeneration, Adsorption

## Abstract

This study addresses the management of sewage sludge (SS), a growing waste worldwide. In the case of the Canary Islands, Spain, 47.3% of the SS is disposed in landfills without further reuse. In this research, SS was used as a precursor to produce activated carbons (ACs), to remove pollutants from wastewater. The ACs were synthesized through chemical activation with phosphoric acid. The carbonization temperature and acid impregnation ratio were optimized, being 650 °C and 2 g H_3_PO_4_·g^−1^ precursor, respectively. The methylene blue (MB) number of the optimized AC (AC-650–2) was 140, and that of a commercial AC used for comparison was 160. Additionally, magnetic activated carbons (MACs) were produced to facilitate material recovery and the regeneration of the adsorbent using an advanced oxidation technique (Fenton). AC-650–2 and MAC-3 adsorbed a maximum of 76.34 mg MB·g^−1^ and 56.76 mg MB·g^−1^, respectively, at 25 °C. The regeneration of the materials was investigated using conventional chemical methods and the Fenton reaction: NaOH washing (for AC-650–2 and MAC) and Fenton (for MAC) enabled recoveries higher than 50% after four cycles. The cost of producing AC-650–2 was estimated at 5.41 €·kg^−1^.

**Graphical Abstract:**

**Figure:**
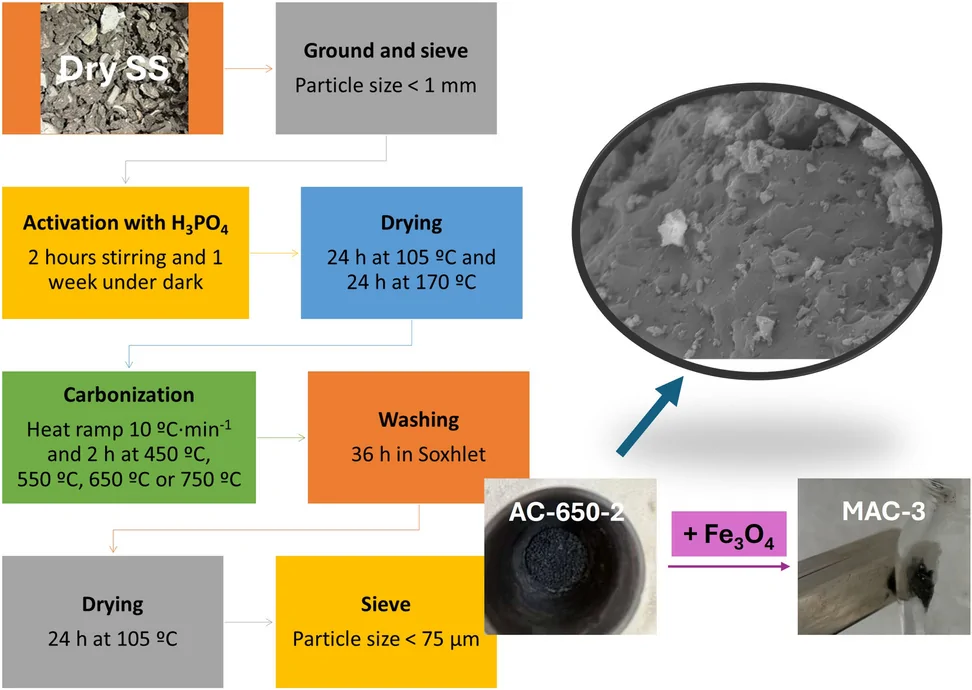

## Introduction

The SS from urban wastewater treatment is increasing due to an increase in population and in WWTP facilities. This residue is normally incinerated or used in agriculture. However, in some regions, like the Canary Islands, these destinations of the SS do not cover the total SS produced. Thus, in this region, 47.3% of the SS is disposed without further reuse or valorization.

It is estimated that, annually, the global population generates 380 billion m^3^ of wastewater, of which only 70% is correctly treated in developed countries, versus 8% in developing countries (Pratap et al. 2023). The treatment of wastewater produces a waste, that is, sewage sludge (SS). The increase in population and wastewater management has inevitably led to an increase in the production of SS (Kelessidis and Stasinakis 2012). In year 2017, globally, 45 million tons of dry SS were produced (Bagheri et al. 2023).

Focusing on our region, in year 2018, over 6.5 million tons of dry SS were produced in in Europe, among which 1.21 million tons were produced in Spain (Eurostat 2022). In year 1986, the European Union (EU) launched the Sewage Sludge Directive (Council Directive 86/278/EEC), that was aimed at the recovery of SS, to stop its discharge into the sea. In year 2014, between 30 and 50% of the SS produced in Europe was used in agriculture, over 20% was incinerated, about 10% was deposited in landfills, about 10% was used for compost, and the rest was disposed (Bianchini et al. 2016). However, this data varies depending on the country, and, within the country, on the region.

For instance, in Spain, in the period 2012–2022, the destination of SS for agriculture increased from 70.8 to 81.7%, about 10% of the SS was incinerated, and the direct disposal of SS in landfills decreased from 17.6 to 10.3%. However, the latter destination still represents a high percentage in some areas at present, such as the Principality of Asturias (78.1%) and the Canary Islands (47.3%) (Instituto Nacional de Estadística 2024).

The composition of SS depends on the pollutant load of the wastewater and on the treatment; it is subjected to in the wastewater treatment plant (WWTP). SS contains suspended and dissolved matter. Some of them have agronomic value, such as organic matter, nitrogen (N), phosphorous (P), as well as other nutrients, including micronutrients. However, SS can also contain contaminants that are hazardous. These contaminants can be inorganic, such as toxic metals, organic, like polycyclic aromatic hydrocarbons (PAHs) or personal care products, among others, and pathogens (Fijalkowski et al. 2017).

Since SS is a carbon-rich resource which is currently being partially disposed, an alternative use of SS could be the production of AC adsorbents, which can be used to remove a wide range of contaminants from air and water. Some previous studies report the fabrication of AC using SS as precursor. Among them, most authors used ZnCl_2_ as activating agent and obtained ACs with surface areas between 300 and 650 m^2^·g^−1^ (Tay et al. 2001a; Zhang et al. 2005; Nunthaprechachan et al. 2013; Djebri et al. 2021; Sanz-Santos et al. 2022; Shi et al. 2022). However, in the last years, the use of ZnCl_2_ as activating agent has decreased, due to its hazardous nature (Martínez-Alvarenga et al. 2024).

Several authors used KOH as activating agent, to produce SS-based ACs with high specific surface areas of up to 1900 m^2^·g^−1^ when the materials were carbonized at high temperatures (700–800 °C) (Monsalvo et al. 2011; Nunthaprechachan et al. 2013; Khoshbouy et al. 2019; Stefanelli et al. 2023; Al-mahbashi et al. 2023). Most authors tested the ACs to remove metal ions from water, although some authors used a dye, methylene blue (MB), to calculate the adsorption capacity of the ACs. In this sense, an adsorption capacity up to 588.2 mg MB·g^−1^ AC was reported for a SS-based AC which was produced using a hydrothermal carbonization (HTC) at 260 °C followed by a chemical activation with KOH and subsequent carbonization under nitrogen atmosphere, at 700 °C for 60 min. The production yield for the AC, defined as the weight ratio between the obtained AC and the precursor, was 17.01% (Khoshbouy et al. 2019). Despite the advantages of using KOH as activating agent to enhance the physico-chemical properties of AC, the use of this corrosive reagent can produce the deterioration of equipment and is also toxic to the environment (Singh et al. 2023). For this reason, recently, the preparation of SS-sludge based AC using a potassium salt, that is KHCO_3_, which is more environmentally friendly, was reported. The resulting AC, carbonized under nitrogen at 800 °C, presented a high specific surface area (952 m^2^·g^−1^), and the adsorption capacity was 162 mg MB·g^−1^ AC (Lu et al. 2023).

Other authors chose acids to activate SS, namely H_2_SO_4_ (Martin et al. 2002; Rozada et al. 2003; Zhang et al. 2005), HNO_3_ (Nunthaprechachan et al. 2013), or H_3_PO_4_ (Zhang et al. 2005; Boualem et al. 2014). Acid-activating agents lead to lower specific surface areas than alkaline ones for SS-based ACs. Namely, the specific surface areas of the mentioned ACs varied between 250 and 410 m^2^·g^−1^ (Martin et al. 2002; Rozada et al. 2003; Zhang et al. 2005; Boualem et al. 2014). The adsorption of MB on a SS-based AC prepared with H_2_SO_4_ (specific surface area 390 m^2^·g^−1^) was only about 20 mg·g^−1^ (Rozada et al. 2003).

Despite this, it must be noted that generally, inert gases, such as nitrogen or argon, are required during the carbonization stage to produce AC when alkaline or neutral activating agents are used. However, this is not necessary when P-group compounds, such as H_3_PO_4_ are used to activate the materials (Gao et al. 2020). This could promote a more environmentally friendly and cheaper production process of the AC if H_3_PO_4_ is used as activating agent instead of others. However, to the best of our knowledge, there are only two previous references in which this activating agent was selected to produce SS-based AC, and none used MB as probe molecule to test the materials. For water treatment, according to the normalized CEFIC test methods for activated carbon, materials should be tested with phenol, iodine, or MB for comparison purposes (European Council of Chemical Manufacturers’ Federations (CEFIC) 1986).

Additionally, AC regeneration is important to increase its life cycle and to improve its use from an environmental and economical point of view. Several regeneration techniques have been reported, such as chemical regeneration, steam regeneration (El Gamal et al. 2018), ultrasonic regeneration (Hong et al. 2020), or advanced oxidation process (AOPs) regeneration (Cabrera-Codony et al. 2015). However, one of the disadvantages of AC is that it must be treated as a hazardous waste at the end of its life, if the adsorbent was used to remove hazardous substances. Among the mentioned regeneration techniques, AOPs are the only methods that enable the possible mineralization of the toxic adsorbates.

AOPs consist of the production of reactive radicals in aqueous solution, mainly hydroxyl radicals (·OH), which oxidize organic compounds. In the case of the regeneration of AC with AOPs, contaminants are transformed on or near the surface of AC by the action of ·OH. Among the AOPs, the Fenton process consists on the reaction of Fe(II) with hydrogen peroxide (H_2_O_2_) to form ·OH. During the Fenton process, the following reactions take place:

1 $${\mathrm{Fe}}^{2+}+{\mathrm{H}}_{2}{\mathrm{O}}_{2}\to {\mathrm{Fe}}^{3+}+\mathrm{OH}+\cdot \mathrm{OH}$$

2 $${\mathrm{Fe}}^{3+}+{\mathrm{H}}_{2}{\mathrm{O}}_{2}\leftrightarrow {\mathrm{FeOOH}}^{2+}+{\mathrm{H}}^{+}$$

3 $${\mathrm{FeOOH}}^{2+}\to {\mathrm{Fe}}^{2+}+\cdot \mathrm{OOH}$$

The hydroxyl radicals (• OH) formed in reaction 1 are highly oxidant and can oxidize the organic matter present in the media. Thus, using this technique, the adsorbate could be mineralized, to recover the adsorbent without producing the contamination of the regeneration solution.

Cabrera-Codony et al. tested several AOPs to recover AC exhausted with siloxane, and demonstrated that, when the AC was impregnated with iron (II) sulfate at pH 2.5, in the presence of H_2_O_2_, the AC recovered up to 92% (Cabrera-Codony et al. 2015). However, another study performed with granular activated carbon (GAC) and dyeing wastewater reported a regeneration efficiency of 50% for the Fenton regeneration process (Chen et al. 2017). A more recent study demonstrated that a GAC containing iron could be recovered by 98.27% up to four times, for the treatment of landfill leachates, with a regeneration method at pH 3 consisting of the addition of H_2_O_2_ (Méndez-Novelo et al. 2023).

Lastly, the combination of AC and magnetic particles like magnetite (Fe_3_O_4_) has been proposed to synthesize magnetic nanocomposites that facilitate the separation and recovery of the material; this combination is commonly named MAC (magnetic AC) (Ali et al. 2023). The separation and complete recovery of magnetic adsorbents can be achieved by applying an external magnetic field. MACs have been used for the removal of dyes and toxic metals, as well as for the remediation of organic contaminants (Rocha et al. 2020). Most studies that report MACs are focused on the fact that there is a great loss of material in the regeneration cycles of powdered AC, and thus, the regeneration efficiencies are limited (Moosavi et al. 2020).

MAC can be regenerated using the Fenton technique due to the high content of iron in the magnetic material. Very few studies have focussed on this topic up to date, to our knowledge. Do et al. reported a 65% regeneration efficiency of MAC for the adsorption of methylene orange (MO) (Do et al. 2011). Other authors used H_2_O_2_ during the adsorption of a pharmaceutical onto MAC, to promote a Fenton-like reaction, and regenerated the MAC with a NaOH solution, finding that the efficiency of the recyclability of the adsorbent decreased by over 16% after 5 cycles (Oruganti et al. 2024). Another recent study revealed that the reaction between Fe_3_O_4_ and oxygen functional groups on the MAC surface with persulfate ions enabled the generation of sulfate radicals, with a high oxidation potential, that promoted the degradation of MO dye and, thus, the regeneration of MAC with 90% efficiency after 5 cycles (Wang et al. 2024).

The aim of this work was, first, to investigate an alternative for the SS that is being currently disposed without further treatment. Namely, in this work SS was used as precursor to fabricate AC. The activating agent chosen was H_3_PO_4_ to avoid using an inert atmosphere in the carbonization step, and, thus, reduce the production costs and technical requirements. The synthesized materials were characterized in terms of chemical composition, and the textural and chemical properties were determined. A dye, methylene blue, was used as probe molecule for adsorption equilibrium and kinetic experiments.

The second aim of this work was to produce a SS-based MAC to enhance an easier recovery or separation of the adsorbent from the treated water, and to study the reusability of the adsorbents after saturation, using several regeneration methods, namely chemical regeneration and regeneration using a Fenton-like reaction. The reusability of the SS-based AC (not magnetic) was also studied.

## Materials and methods

### Activated carbon preparation

The SS precursor was taken from a WWTP in Gran Canaria. The SS was dried in drying beds in the WWTP facility. Next, it was grounded and sieved, and the particles smaller than 1 mm were impregnated with a solution of H_3_PO_4_ (85% purity, Panreac) for 1 week and were next dried at 105 °C for 24 h, and at 170 °C for another 24 h, before being carbonized for 2 h at a given temperature. Next, the materials were washed with water in a Soxhlet extractor for 36 h. Finally, the resulting ACs were dried at 105 °C for 24 h and sieved to ensure particle sized lower than 75 µm. The ACs were named as follows: *AC-T-A*, being *T* the carbonization temperature (450, 550, 650, and 750 °C) and *A* the activating agent concentration (0.7 or 2 g H_3_PO_4_·g^−1^ precursor).

Using the optimum AC, the MAC material was synthesized by the co-precipitation method, using the Massart’s method to produce Fe_3_O_4_ (Massart 1981). For this purpose, the following were mixed: 40 mL of 1 M FeCl_3_, 10 mL of 2 M FeCl_2_ (prepared in a solution of HCl, 2 M), 500 mL of 0.7 M NH_3_ and *x* g of AC, being × 0.2, 0.6, 1, or 3 g. This mixture led to the instant formation of Fe_3_O_4_ over the AC. The resulting mixture was left to settle and washed thoroughly with distilled water. Next, the material was left to dry in the oven at 80 °C. The resulting MACs were named as follows: *MAC-x.*

## Characterization of the materials

Thermogravimetric analysis (TGA) and differential scanning calorimetry (DSC) of the precursor and activated precursor was performed in a TA Instruments Discovery SDT 650 Simultaneous Thermal Analyzer (TGA/DSC). Samples were analyzed between ambient temperature and 900 °C under air, with a heating rate of 10 °C·min^−1^.

The yield of production, *η*_*p*_, was calculated for each material following Eq. 1. To determine the mass, a Sartorius Entris balance with a precision de 0.1 mg was used. The yield after the carbonization stage, *η*_*c*_, before the washing step, was also calculated using Eq. 4, but the mass of AC was replaced in the equation by the mass of material after carbonization.

4 $${\eta }_{p} \left(\%\right)=\frac{\text{mass of AC}}{\text{mass of precursor} }\cdot 100$$

Ash content of the ACs and MAC was determined following standard ASTM D2866-11. Briefly, the materials were left to dry at 150 °C for 3 h to eliminate humidity, and, subsequently, were subjected to 650 °C for 3 h. The ash content was calculated from the mass of ash that remained after this process.

The pH at the point of zero charge, pHpzc, was measured following (Rivera-Utrilla et al. 2001). Briefly, the pH of several 0.01 M NaCl solutions was adjusted (Crison micropH 2002 pHmeter) between 2 and 10 by adding small volumes of 0.1 M HCl and 0.1 M NaOH. Subsequently, 0.075 g of the material was added to each solution and left in an orbital shaker at 100 rpm and 25 °C for 24 h. After this, the final pH of each solution was measured. The initial vs final pH was plotted, and the pHpzc was obtained from the intersection of the curve with the bisector of the graph.

Elemental analysis was used to determine the proportion of carbon, nitrogen, hydrogen, and sulfur (C, H, N, S) present in the materials. The percentage of oxygen was calculated from Eq. 5. The equipment used for these measurements was the elemental analyzer Thermo Scientific FlashEA 1112.

5 $$\%\;\mathrm{O}=100-\% \mathrm{C}-\% \mathrm{H}-\% \mathrm{N}-\% \mathrm{S}$$

X-ray fluorescence (XRF) was used to determine the presence of other elements in the materials. For this purpose, a Bruker S2 Puma energy dispersive X-ray fluorescence spectrometer was used.

The porosity of the materials was determined by nitrogen adsorption–desorption isotherms at − 196ºC using a Micro 300 3P INSTRUMENTS equipment. The samples were degassed using a 3P prep J4 Degasser at 150 °C for 12 h under vacuum prior to physisorption analysis. The specific surface area was calculated following the Brunauer-Emmet-Teller (BET) method, the total volume at relative pressure of 0.99, the mesopore volume with the BJH method, and the micropore volume with *t*-plot graph, both of them with the Broekhoff-De Boer *t*-curve.

A scanning electron microscope (SEM), Hitachi TM3030, equipped with energy dispersive X-ray spectrometer (EDX) was used to observe the surface morphology of the samples. The samples were sputter-coated with gold for charge dissipation before analysis.

### Adsorption studies

#### MB value

The synthesized ACs were subjected to the MB test, as specified in the MB value standard of CEFIC (European Council of Chemical Manufacturers’ Federations (CEFIC) 1986)*.* The MB test is widely used in the characterization of ACs as it gives an estimate of the adsorption capacity of an AC, for molecules with similar dimensions to the MB. This test was also performed on a commercial AC (Panreac no. 121237) for comparative purposes. Once the test was carried out, the AC with highest MB value was selected for further studies.

For this purpose, first, a 1200 mg·L^−1^ MB solution was prepared. Then, the test solution was prepared by taking 5 mL of the latter solution and diluting it to 1000 mL in a volumetric flask with a 0.25% (v/v) acetic acid solution. Next, 0.1 g of the carbon sample was added to 25 mL of the MB test solution in a flask and left to stand for 5 min. After that time, a 1 mL sample was taken from the flask and centrifuged to determine if the MB test solution was decoloured. Additional samples of 5 mL of the test solution were added into the flask until the AC became saturated, that is, until no discoloration of the sample was observed after 5 min. Finally, the total volume of MB that the AC was able to adsorb was registered as the MB number.

#### Iodine number

The iodine number is also used to determine the capacity of ACs, mainly associated with microporosity. Iodine number was determined according to Standard AWWA B 600–78 for the AC that presented the highest MB values, as well as for the commercial AC.

To determine the iodine number, 1.5 g of AC was poured into 10 mL of a 5% HCl solution, and the mixture was left to boil for 30 s. Next, after the content cooled, 100 mL of a standardized 0.1 N iodine solution was added, and the mixture was then filtered to collect 50 mL that were titrated with a 0.1 N sodium thiosulfate solution in presence of starch, until the blue color disappeared. This allowed to calculate the mass of iodine adsorbed by the activated carbon, in mg iodine·g^−1^ AC.

#### Adsorption equilibrium experiments

Adsorption equilibrium studies were carried out with several 100 mL samples of a 50 mg·L^−1^ MB solution at natural pH and at a constant temperature of 25 °C, following standard ASTM D3860-98. Different adsorbent loads were tested, between 10 mg and 4 g. The samples were subjected to orbital shaking at 100 rpm for 24 h to ensure that the adsorption equilibrium was reached. Afterwards, the MB concentration was measured spectrophotometrically at 664 nm in a Perklin Elmer Lambda 2 spectrophotometer. The detection and quantification limits for MB were 0.26 and 0.75 mg·L^−1^, respectively. The adsorption capacity of the ACs at equilibrium, *q*_*e*_ (mg·g^−1^), was calculated following Eq. 6,

6 $${q}_{e}=\left(\frac{{C}_{0}-{C}_{e}}{m}\right)\times V$$

being *C*_0_ (mg·L^−1^) the initial concentration of MB, *C*_*e*_ (mg·L^−1^) the concentration of MB at the adsorption equilibrium, *m* (g) the mass of AC, and *V* (L) the volume of solution.

Several mathematical models can be employed to study adsorption isotherms, such as Langmuir, Freundlich, and Temkin. In this work, the first two were chosen, because they are the most widely used in the literature for MB adsorption (Björklund & Li 2017; Chen et al. 2002; Lu et al. 2023; Tay et al. 2001a, b).

The Langmuir model describes that the adsorption occurs on a homogeneous surface through the formation of a monolayer, and with no interaction between the adsorbed molecules. The model follows Eq. 7 (Langmuir 1918).

7 $${q}_{e}=\frac{{q}_{\mathrm{mon}}\cdot {K}_{\mathrm{L}}\times {C}_{e}}{1+{K}_{\mathrm{L}}\times {C}_{e}}$$

where *q*_*e*_ (mg·g^−1^) is the adsorption capacity, *q*_mon_ (mg·g^−1^) is the maximum adsorption capacity to complete the monolayer, *K*_L_ (L·mg^−1^) is the Langmuir constant, and *C*_*e*_ (mg·L^−1^) is MB concentration at the adsorption equilibrium.

In addition, the separation factor, *R*_L_, can be calculated from the Langmuir constant, following Eq. 8, to determine the type of adsorption that occurred (Zawrah et al. 2023):

8 $${R}_{L}=\frac{1}{1+{K}_{L}\times {C}_{0}}$$

In Eq. 8, *C*_0_ is the initial adsorbate concentration in mg·L^−1^. When RL > 1 adsorption is unfavorable. R_L_ = 0 indicates an irreversible adsorption, RL = 1 corresponds to a linear adsorption; and 0 < RL < 1 is a favorable adsorption.

The Freundlich isotherm is an empirical model that proposes the non-ideal adsorption on a heterogeneous surface by the formation of multilayers, and with interaction between the molecules. This model follows Eq. 9 (Van der Bruggen 2015).

9 $${q}_{e}={K}_{\mathrm{F}}\times {{C}_{e}}^\frac{1}{n}$$

where *K*_F_ (mg·g^−1^) (L·mg^−1^)^1/*n*^ is the Freundlich constant, and 1/*n* is the adsorption intensity. If the value of 1/*n* is between 0 and 1, the adsorption is favorable (Medina et al. 2014).

In this work, the parameters of the Langmuir and Freundlich models were calculated using the non-linear method.

#### Adsorption kinetic experiments

The adsorption kinetics were studied in two different water matrices (tap water and distilled water), to determine the effect of the ionic strength of the water matrix. Likewise, adsorption was studied at different pH values. Namely, experiments were done at pH 4 and 8.

The kinetic studies were only done with the optimum ACs and MAC. For these experiments, 0.1 g activated carbon was poured into 100 mL of a 50 mg·L^−1^ MB solution. The solution was kept in an orbital stirrer at 25 °C, and samples were taken every 2 min for the first 10 min, then every 5 min until 30 min, and every 10 min after that time. The MB concentration was determined for each sample to obtain the adsorption capacity of the activated carbon with contact time.

The adsorption capacity at each contact time, *q*_*t*_ (mg·g^−1^), was calculated following Eq. 6, by replacing *C*_*e*_ with C_*t*_ (mg·L^−1^), that is, the concentration of MB at each time.

There are several kinetic models for adsorption, among which the pseudo-first-order model and the pseudo-second-order model are most frequently used in the study of ACs fabricated from SS (Bezerra et al. 2019; Lu et al. 2023).

Lagergren’s pseudo-first-order model (PFO) follows Eq. 10 (Tseng et al. 2010):

10 $${q}_{t}= {q}_{e}(1-{e}^{{-k}_{1}t })$$

where *q*_*t*_ (mg·g^−1^) is the adsorption capacity at each time (t) and *k*_1_ (min^−1^) is the pseudo-first adsorption constant.

Pseudo-second-order model (PSO) follows Eq. 11 (Ho and Mckay 1999):

11 $${q}_{t}= \frac{{k}_{2}{q}_{e}^{2}t}{1+{k}_{2}{q}_{e}t}$$

where *k*_2_ (g· mg^−1^·min^−1^) is the pseudo-second adsorption constant.

In this work, the parameters of the kinetic models were calculated using the non-linear method.

The adsorption process was further investigated with the Weber and Morris intraparticle diffusion model (Weber & Morris 1964), which follows Eq. 12:

12 $${q}_{t}= {k}_{i}{t}^{0.5 }+{c}_{i}$$

where *k*_*i*_ (mg·g^−1^·min^−0.5^) is the intraparticle diffusion rate constant and *c*_*i*_ (mg·g^−1^) is the constant related to the thickness of the boundary layer.

#### Adsorption thermodynamics

For the thermodynamic analysis of the adsorption of MB onto the activated carbon, adsorption equilibrium experiments were done at 10 °C, 25 °C, and 40 °C. The distribution coefficient of adsorption, *K*_*d*_, was calculated from the intercept of the plot of ln(*q*_*e*_/*C*_*e*_) versus *q*_*e*_, as described elsewhere (Lyubchik et al. 2004). Next, the Gibbs free energy change (Δ*G*, J·mol^−1^) was determined from the Eq. 13. Subsequently, entropy change (Δ*S*, J·mol^−1^·K^−1^) and enthalpy change (Δ*H*, J·mol^−1^) were calculated form the slope and the intercept, respectively, of the plot of Δ*G* versus *T*, as depicted from Eq. 14.

13 $$\Delta G=-\mathrm{RT}ln{K}_{d}$$

14 $$\Delta G=\Delta H-T\Delta$$

where *R* is the universal gas constant (8.314 J·mol^−1^·K^−1^) and *T* is the temperature (K).

#### Reuse and regeneration studies

Different reuse methods were studied for the optimal AC and MAC. The material (1 g·L^−1^) was first saturated with 100 mL of a 50 mg·L^−1^ MB solution at pH 8, prepared with tap water. Next, the AC was recovered by centrifugation, and washed using 0.1 M HCl, 0.1 M NaOH, 70% v/v ethanol, or water at pH 2 (adjusted with HCl). For this, the materials were shaken in the abovementioned washing solutions for 24 h at 25 °C in an orbital shaker. Once this time had elapsed, the materials were washed and centrifugated with small volumes of distilled water to remove excess acid, base or ethanol. Finally, the regenerated materials were used again to adsorb 100 mL of a 50 mg·L^−1^ MB solution at pH 8.

Likewise, the regeneration of MAC was also carried out with H_2_O_2_ at pH 2 (adjusted with HCl). The possible lixiviation of iron from MAC could react with H_2_O_2_ to regenerate the material through the Fenton reaction.

In all cases, the evolution of MB adsorption was followed for several adsorption cycles. For the experiments with MAC, additionally, the concentration of iron and H_2_O_2_ was determined spectrophotometrically.

The determination of total iron in solution was done using the o-phenanthroline method (Saywell and Cunningham 1937). The determination was done by adding 2.5 mL of concentrated sulfuric acid, H_2_SO_4_, 2 mL of a 10% wt. hydroxylamine hydrochloride solution, 10 mL of a 0.1 M sodium acetate solution, and 5 mL of a 2·10^−3^ M 1,10-phenanthroline solution to a 50-mL volumetric flask, that was finally leveled off with the sample. Hydroxylamine hydrochloride reduces Fe(III) to Fe(II), and Fe(II) forms a red complex with 1,10-phenanthroline, which can be followed spectrophotometrically at 511 nm. The quantification limit was 0.25 mg·L^−1^.

To quantify the concentration of H_2_O_2_ in solution, 2.5 mL of a 0.38 M H_2_SO_4_ solution and 2 mL of a 50 g·L^−1^ potassium titanium (IV) oxalate solution were mixed and made up to 25 mL with the sample. The yellow complex formed was measured spectrophotometrically at 411 nm (Sellers 1980). The quantification limit was 0.1 mg·L^−1^.

The energy requirements to synthesize the materials at laboratory scale were measured using a Lexman F-type digital socket energy monitoring.

## Results and discussion

### TGA/DSC analysis

**Fig. 1:**
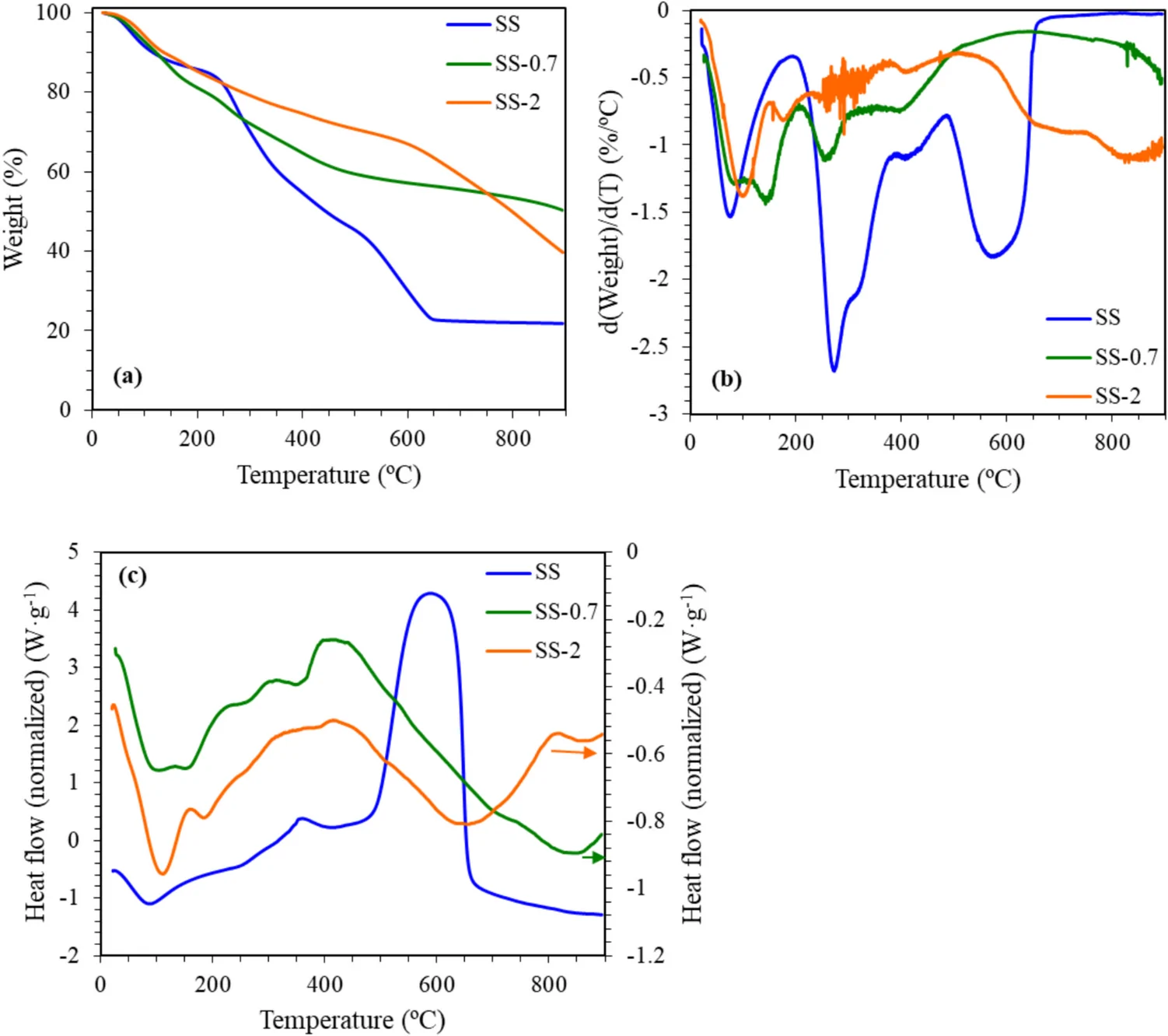
**a** Thermogravimetric analysis curve (TG), **b** first derivative of the weight loss (DTG), and **c** differential scanning calorimetry (DSC) results for the SS and the SS impregnated with 0.7 (SS-0.7) or 2 (SS-2) g H_3_PO_4_·g SS

TGA and DSC analysis results for the SS precursor and the SS impregnated with H_3_PO_4_ are shown in Fig. 1. Namely, sample SS-0.7 corresponds to the SS impregnated with 0.7 g H_3_PO_4_·g^−1^ precursor, and SS-2 is the SS sample impregnated with 2 g H_3_PO_4_·g^−1^ precursor.

The heat flow (Fig. 1c) for the SS presented positive values, which indicated that the process was endothermic, from 300 to 600 °C. The highest value was observed at 587 °C. The endothermic process indicated the degradation of organic matter in the SS. This observation is in line with that reported by another author (Pavlík et al. 2016).

From the weight loss and its derivative (Fig. 1a and b), several stages were observed for the SS analysis. First, between 0 and 250 °C, humidity was removed (Boualem et al. 2014). At this stage, about 20% mass was lost in all materials (SS, SS-0.1, and SS-2). Next, between 200 and 300 °C, about 45% of the initial mass was lost in the SS. Here, biodegradable and semi-volatile matter were removed. Between 300 and 450 °C, an additional 20% mass loss was observed, which can be attributed to the degradation of organic polymers present in the SS. Lasty, above 450 °C, non-biodegradable matter was removed (Hernandez et al. 2015), which account for an additional 20% mass loss. In summary, 78.2% of the initial SS mass was lost during the heating process under air and up to 900 °C.

The weight loss curve (Fig. 1a) obtained for both activated SS was significantly different to that of the SS. Specifically, between 200 and 450 °C, only 10 and 20% mass loss was observed for SS-2 and SS-0.7, respectively. The mass loss for the SS during this temperature range was much higher (45%). It is known that H_3_PO_4_ dehydrates and forms polyphosphoric acids, such as H_4_P_2_O_7_ y H_5_P_3_O_10_, above 200 °C (Wang et al. 2008). It has been also reported that H_3_PO_4_ forms phosphate bonds that cross-link with each other, leading to polyphosphate bridges that stabilize the organic matter (Zubir and Zaini 2020) and, thus, the lower mass loss registered for the SS impregnated with the highest concentration of H_3_PO_4_ can be related to this. However, due to the breakdown of the phosphate bridges and the consequent evaporation of the polyphosphoric acids above 600 °C, the SS-2 sample presented a higher mass loss than SS-0.7 between 600 and 900 °C (Wang et al. 2008). Globally, 50 and 60% of the initial mass was lost for SS-0.7 and SS-2 at 900 °C.

### Production yields and ash content

The yield after the carbonization stage, *η*_*c*_, and the yield of production, *η*_*p*_, are depicted in Table 1 for each of the ACs synthesized in this work. It must be noted that these findings cannot be compared with literature, because, to the best of our knowledge, no other authors report carbonization yields for ACs fabricated from SS using a self-generated atmosphere in the carbonization stage. However, when comparing with other studies in which AC was synthesized from SS using different activating agents and an inert atmosphere in the carbonization stage, the yield after carbonization ranges from 38.5 to 74.9% (Martin et al. 2002), which is in the range of the results obtained in this work. As expected, the lower yields were obtained, both in literature and in this study, for the ACs carbonized at higher temperatures.

**Table 1:** Yield after the carbonization (η_*c*_) and washing (η_*p*_) stages. Ash content

ACs	η_*c*_ (%)	η_*p*_ (%)	Ash content (%)
AC-450–0.7	64.00	26.17	28.40
AC-550–0.7	58.57	27.13	47.40
AC-650–0.7	54.70	17.37	50.12
AC-750–0.7	44.68	13.04	48.00
AC-450–2	78.80	18.03	31.26
AC-550–2	66.00	15.10	52.52
AC-650–2	59.75	13.78	46.67
Commercial AC	-	-	9.40

On the other hand, we observed that the ACs produced with a higher activating agent dose presented higher production yields. This is in line with the TGA results (see Fig. 1), which concluded that a higher impregnation ratio led to a lower mass loss at a given carbonization temperature.

Similarly, the ash content increased with carbonization temperature. The ash content determined in this study (28.40–52.52%) is in line with those reported by other authors that fabricated ACs from SS, which varied from 37.39% (Tay et al. 2001b) to 53.20% (Almahbashi et al. 2021). It is suggested that ash content can be reduced by acid washing, which would dissolve the inorganic components (Smith et al. 2009). A high ash content can indicate the presence of compounds containing Ca, Al, Mg, Si, or Fe, among others, which are common and can represent up to 50% of the composition of SS, as reported by other authors (Li and Jiang 2017; Pancevska and Zendelska 2022).

It is important to highlight that the yield after washing is not entirely reliable, as the washing process may lead to material losses, mainly due to the drag of AC particles by water during each renewal.

### Chemical characterization

From pH_PZC_ results, we conclude that the materials were of acidic nature, which was expected due to the use of H_3_PO_4_ as activating agent. The commercial AC also presented an acidic pH_PZC_.

The CHNS elemental composition of the SS and the synthesized ACs, as well as their pH_PZC_ is shown in Table 2. The carbon content is much higher in the commercial AC compared to the SS-based ACs. Similar C wt.% compositions are reported in most articles in which AC was manufactured from SS (Chen et al. 2002; Boualem et al. 2014; Almahbashi et al. 2021). For the ACs synthesized at the lowest carbonization temperature, the AC contained a higher C wt.% than the SS used as precursor. In general, higher carbonization temperatures led to lower C wt.% and to a decrease in H, N, and S. The decrease in carbon content with increasing temperature can be due to the removal of organic volatile compounds at the carbonization stage.

**Table 2:** CHNS elemental composition and pH_PZC_ of the SS and ACs

Material	% C	% H	% N	% S	% O^a^	pH_PZC_
SS	34.54	7.61	5.58	0.99	51.28	-
AC-450–0.7	40.59	4.76	3.67	0.20	50.78	3.15
AC-550–0.7	23.29	2.12	1.61	0.07	72.91	3.00
AC-650–0.7	30.79	2.48	2.00	0.09	64.64	3.10
AC-750–0.7	22.69	1.99	1.33	0.16	73.83	-
AC-450–2	39.13	4.58	3.40	0.28	52.61	2.50
AC-550–2	32.27	3.18	1.98	0.12	62.45	3.01
AC-650–2	24.94	0.61	2.40	-	72.05	3.00
Commercial AC	88.00	0.50	0.50	-	11.00	3.00

^a^Calculated by difference

All materials presented a high oxygen content, and, in general, it increased with carbonization temperature and with the H_3_PO_4_: precursor impregnation ratio. The high oxygen content can be related to the high organic matter present in the SS and to the addition of H_3_PO_4_ (Boualem et al. 2014). In addition, the carbonization in a self-generated atmosphere, where air is present, can lead to the oxidation of carbon, and thus, a higher oxygen content in the resulting materials (Martín-González et al. 2014).

As for the low content of H and N in the ACs with respect to the sludge, it can be attributed to the release of these in the carbonization process: the higher the temperature, the higher their release (Demiral et al. 2021).

On the other hand, it is known that the presence of toxic metals and emerging contaminants, such as pharmaceuticals in SS, is an inconvenient to its reuse (Geng et al. 2020). To determine the content of toxic metals and other elements, XRF analysis was performed to the SS, and one of the ACs fabricated in this work, namely is AC-650–2. Results are shown in Table 3.

**Table 3:** XRF analysis of SS and AC-650–2. The results shown correspond to the weight percentage of each element in the material

Element (wt. %)	Material	
	SS	AC-650–2
**Mg**	1.72	0.22
**Al**	6.04	2.88
**Si**	19.36	17.49
**P**	11.63	67.21
**S**	8.70	1.54
**Cl**	2.02	0.82
**K**	4.82	-
**Ca**	23.36	-
**Ti**	4.48	2.54
**Mn**	0.53	-
**Fe**	15.22	7.30
**Zn**	2.13	-

Notably, inorganic elements such as Ca, Mg, Al, Si, and Fe and nutrients such as N, P, and K, were present in the SS precursor, as expected (Li and Jiang 2017). Ca was the most abundant element in the SS, maybe due to the use of salts containing iron and lime to reduce P concentrations in wastewater, to meet regulatory standards (Shrivastava et al. 2022). Among the mentioned elements, K and Ca were not present in the AC; and Mg, Al, S, and Fe were considerably reduced during the synthesis of AC. This confirms that washing the ACs with water is enough to remove most of the soluble ash fractions.

However, Si was only slightly reduced in the AC. It is known that Si forms SiO_2_, which can react with H_3_PO_4_ producing SiP_2_O_7_. If not removed, this compound can accumulate in the pores of the AC, leading to a reduction in surface area and pore volume (Li et al. 2011). In this, sense, as mentioned above, acid and/or base refluxes could improve the removal of inorganic substances from the AC. Specifically, other authors reported several acid/basic washing processes for SS-based AC, and found that the best procedure to remove SiO_2_ from the AC was to wash the material with a 1 N HCl solution and next introduce it in an autoclave with 1 N HF (Aliakbari et al. 2018). However, in this study, this was not done to maintain the synthesis process simple and cost-effective.

It can be observed that the amount of P is higher in the AC than in the SS. This is due to the use of H_3_PO_4_ as an activating agent and indicates that the washing process did not remove all the phosphorus introduced during the activation stage.

The SS presented toxic metals, Mn and Zn, in its composition, which were removed during the synthesis of the AC, and, thus, were not present in the AC. These elements, together with K and Ca, were most probably removed during the washing stage after the materials were carbonized. It must be noted that the EU establishes the maximum concentration of toxic metals in SS accepted for the use of this residue in agriculture (Council Directive 86/278/EEC). Such, the content of Zn must be limited to 300 mg·kg^−1^ of dry sludge. The concentration of Zn found in the SS used in this study was 21,300 mg·kg^−1^, which surpassed the legal limit if the SS was to be used in agriculture. For this reason, an alternative use, such as the one presented in this paper, must be provided to reuse the SS used in this work.

### Textural characterization

Table 4 shows the parameters obtained from the BET surface area, total volume (*V*_*t*_), mesopore and micropore volume (*V*_mes_ and *V*_micro_), and average pore diameter (*d*_*p*_) for each of the ACs, including the magnetic ones, as well as for the commercial AC, the SS, and Fe_3_O_4_.

**Table 4:** BET, *V*_*t*_, *V*_mes_, and *d*_*p*_ surface areas of SS, Fe_3_O_4_, and commercial and synthesized ACs and MACs

Material	BET (m^2^·g^−1^)	*V*_*t*_ (cm^3^·g^−1^)	*V*_mes_ (cm^3^·g^−1^)	*V*_micro_ (cm^3^·g^−1^)	*D*_*p*_ (nm)
SS	87.24	0.109	0.130	0.000	4.99
AC-450–0.7	358.56	0.352	0.224	0.051	3.93
AC-550–0.7	369.10	0.359	0.226	0.058	3.89
AC-650–0.7	256.64	0.223	0.125	0.056	3.48
AC-750–0.7	167.65	0.179	0.138	0.006	4.28
AC-450–2	579.07	0.597	0.429	0.058	4.13
AC-550–2	325.93	0.373	0.301	0.009	4.57
AC-650–2	227.14	0.238	0.156	0.031	4.20
MAC-0.2	223.39	0.607	0.569	0.001	9.26
MAC-0.6	289.98	0.514	0.368	0.016	7.09
MAC-1	275.33	0.412	0.287	0.017	5.99
MAC-3	429.66	0.573	0.508	0.026	5.34
Commercial AC	1022.95	0.473	0.081	0.381	1.85
Fe_3_O_4_	68.61	0.305	0.255	0.0	17.78

The synthesized ACs presented much higher specific surface areas and mesoporosities than the SS used as precursor, which had a specific surface area equal to 87.24 m^2^·g^−1^. In fact, the specific surface areas of the ACs fabricated in this work were higher than those reported in some other references in which SS was used as precursor (Zhang et al. 2005; Hunsom and Autthanit 2013; Boualem et al. 2014). An increase in carbonization temperature dealt to a lower specific surface area, in agreement with that reported by other authors (Tay et al. 2001a; Navarro and Vargas 2010).

For the materials synthesized with lower loads of activating agent, the pore volume was almost constant for the ACs carbonized between 450 and 550 °C; at higher temperatures it decreased, and the low microporosity of the materials fabricated at 750 °C led to a notable decrease in the total pore volume. The materials fabricated with the higher concentration of activating agent presented higher total pore volumes (i.e., 0.597 cm^3^·g^−1^ at 450 °C), but these decrease with increasing carbonization temperatures. The materials AC-650–0.7 and AC-650–2 exhibit very similar textural properties, which suggests that, at this carbonization temperature, the temperature effect prevails over the concentration of activating agent. This does not occur at lower carbonization temperatures, where differences are observed for the materials fabricated with different activating agent loads. Additionally, an increase in *d*_*p*_ was observed as the impregnation ratio of the activating agent increased, which can be explained by the widening of the pores caused by this increase, as noted in another reference (Boualem et al. 2014).

Regarding the MACs, an increase in the surface area was noticeable as the amount of AC used in the synthesis of the material increased. These surface areas were higher than those of AC-650–2, which was the AC used for the synthesis of the MACs. Similar results were observed by other authors (Altıntıg et al. 2017; Hao et al. 2018).

**Fig. 2:**
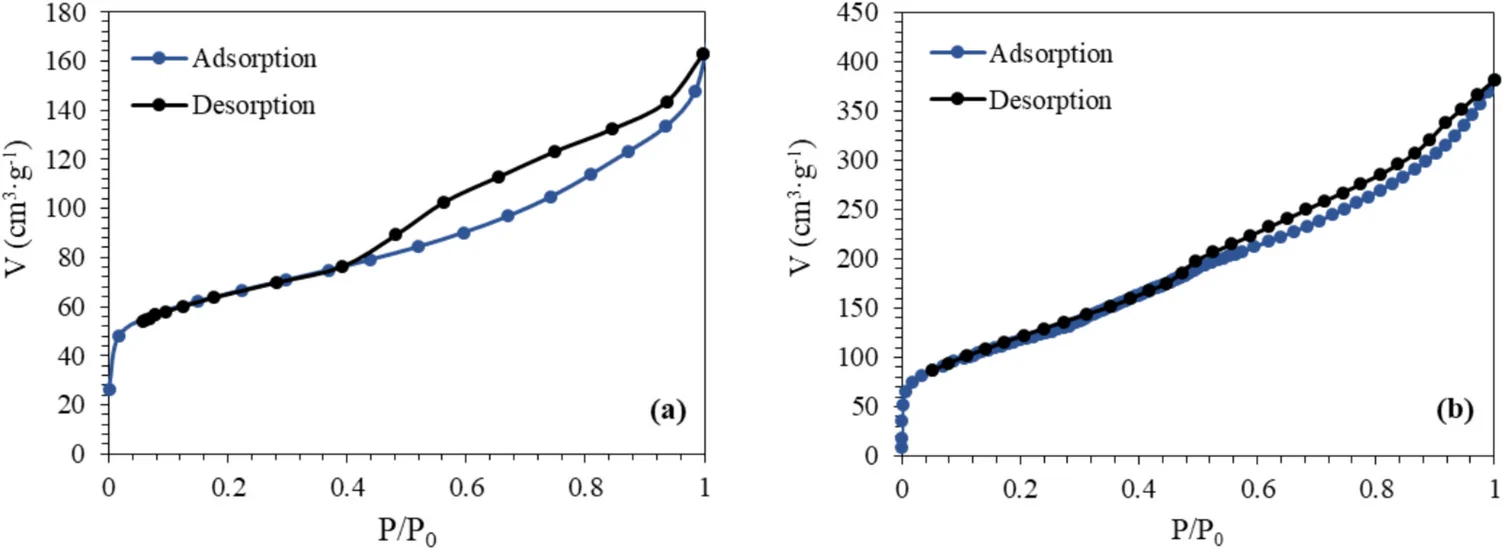
Nitrogen adsorption–desorption isotherms for **a** AC-650–2 and **b** MAC-3

Figure 2 shows the nitrogen adsorption–desorption isotherms for two materials: AC-650–2 and MAC-3. The isotherms were like type IV of the IUPAC classification, which suggests that the materials are mainly mesoporous (Allen et al. 1998). This same type of isotherm has been reported in literature for other SS-based ACs (Boualem et al. 2014).

**Fig. 3:**
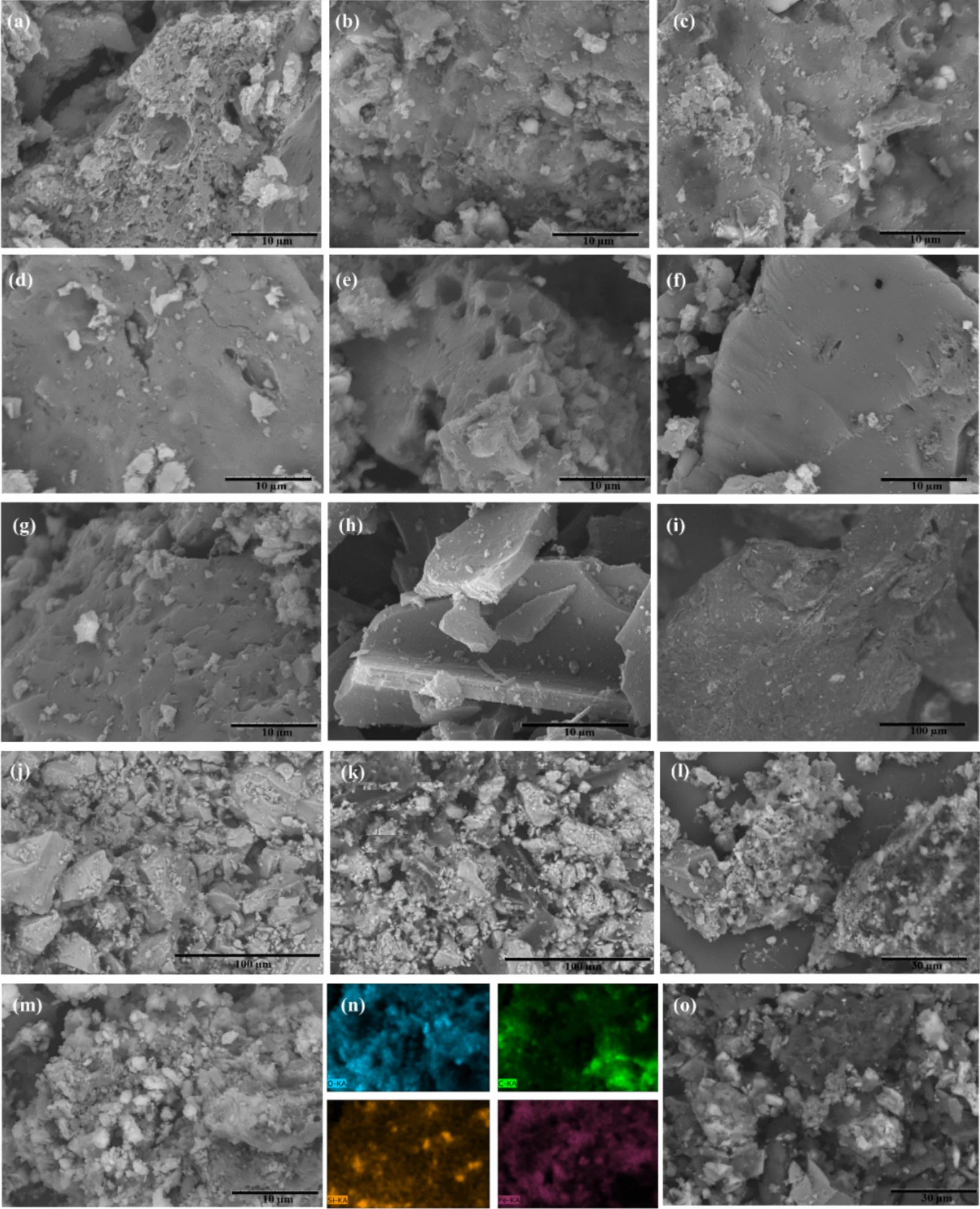
SEM micrographs of **a** AC-450–0.7, **b** AC-550–0.7, **c** AC-650–0.7, **d** AC-750–0.7, **e** AC-450–2, **f** AC-550–2, **g** AC-650–2, **h** commercial AC, **i** SS, **j** Fe_3_O_4_, **k** MAC-1 (synthesized using commercial AC), **l** MAC-0.6, **m** MAC-1, **n** EDS of MAC-1, and **o** MAC-3

Figure 3 shows the SEM micrographs of the materials. In general, for all the synthesized materials, the shape of the particles was irregular.

It is clearly observed that the surface of the ACs activated with a lower concentration of activator (see Fig. 3a-d) had a less modified and a rougher surface, like that of the original SS. However, in the ACs obtained with a higher dose of activator (see Fig. 3e-g), the greater chemical attack of phosphoric acid was clearly detected, resulting in a texture that differed from that of the original precursor, and, in addition, the porosity formed was more clearly observed.

In this sense, as depicted in Table 4, at equal carbonization temperatures, higher activating agent concentrations led to materials with higher total pore volumes and mesopore volumes, especially at the lower temperatures.

From EDS analysis (not shown), the main element found in the surface of the ACs fabricated in this work was carbon (C), followed by oxygen (O). Small amounts of phosphorous (P), iron (Fe), silicon (Si), and aluminum (Al) were also detected. This agrees with the XRF results shown in Table 3. Similarly, calcium (Ca) was detected by EDS in the SS sample but not in the ACs, which is in line with the XRF results. EDS analysis revealed that the content of C in the SS was 47.26% while the C content in the ACs was in the range of 56–81%.

Regarding MACs, a good distribution of Fe_3_O_4_ particles was observed over the activated carbon for all the MACs, despite of the greater or lower carbon content in the magnetic material (see Fig. 3k-o).

### Batch adsorption studies

The optimum AC and MAC were chosen based on the materials that exhibited the highest MB number, that indicated their greater adsorption capacity. Table 5 shows the MB number found for each of the ACs and MACs, compared to the commercial AC and MAC-1 prepared using commercial AC. The synthesized materials that presented the highest adsorption capacities were AC-650–2 and MAC-3, with MB numbers of 140 mL and 80 mL, respectively. The MB numbers obtained for these materials were only slightly lower than those of the commercial AC (160 mL) and the MAC-1 synthesized using commercial AC (85 mL). Experiments were also done with the precursor alone (SS) and Fe_3_O_4_ (synthesized as described in the “Activated carbon preparation” section but without AC), for comparison purposes. The MB adsorption capacity of both materials was negligible.

**Table 5:** MB number for the ACs and MACs

Material	MB number (mL)	Iodine number (mg·g^−1^)
AC-450–0.7	85	-
AC-550–0.7	110	-
AC-650–0.7	135	-
AC-750–0.7	110	-
AC-450–2	130	-
AC-550–2	100	-
AC-650–2	140	682.50
Commercial AC	160	821.34
MAC-0.2	45	-
MAC-0.6	60	-
MAC-1	70	-
MAC-3	80	531.52
MAC-1 (from commercial AC)	85	712.08

The different activating agent loads only produced a noticeable difference in the MB number results at a carbonization temperature of 450 °C. As mentioned above, the lower concentration of activator, 0.7 g H_3_PO_4_·g precursor, at low carbonization temperature, was not enough to produce an important textural change in the resulting activating carbon, compared to the SS used as precursor.

The iodine number was determined for the materials that exhibited a higher MB number. Results are shown in Table 5. The iodine number was lower for the synthesized ACs than for the commercial AC, which is consistent with the higher microporosity of the latter. Additionally, the high content of metallic oxides in the synthesized ACs, because of the precursor used, resulted in a repulsion with I_3_^−^. MACs exhibit even lower iodine numbers due to the presence of Fe_3_O_4_ over the activated carbon. Other authors, that synthesized SS-based ACs using different methods to that reported in this study, informed iodine numbers which are in line with those reported in this work.

For example, Pancevska et al. reported an iodine number of 571.3 mg·g^−1^ for a SS-based AC prepared with ZnCl_2_, using a 2:1 precursor: ZnCl_2_ proportion for activation, and then carbonized at 600 °C (Pancevska and Zendelska 2022b). Rozada et al. used a 1:1 precursor: ZnCl_2_ proportion and a carbonization temperature of 650 °C and achieved an iodine number of 962.7 mg·g^−1^ (Rozada et al. 2005). The same authors reported a lower iodine number (535.7 mg·g^−1^) for the SS-based AC, when the SS was activated with sulfuric acid under the same conditions (Rozada et al. 2005). Thus, the activating agent chosen in the synthesis procedure has demonstrated to influence on the iodine adsorption capacity of ACs. Wang et al. concluded that higher iodine numbers were obtained for SS-based ACs fabricated using KOH (864.8 mg·g^−1^), compared to others synthesized using ZnCl_2_ and KCl (Wang et al. 2009).

#### Adsorption isotherms

Adsorption isotherms were studied for the optimum AC and MAC, that is, AC-650–2 and MAC-3.

**Fig. 4:**
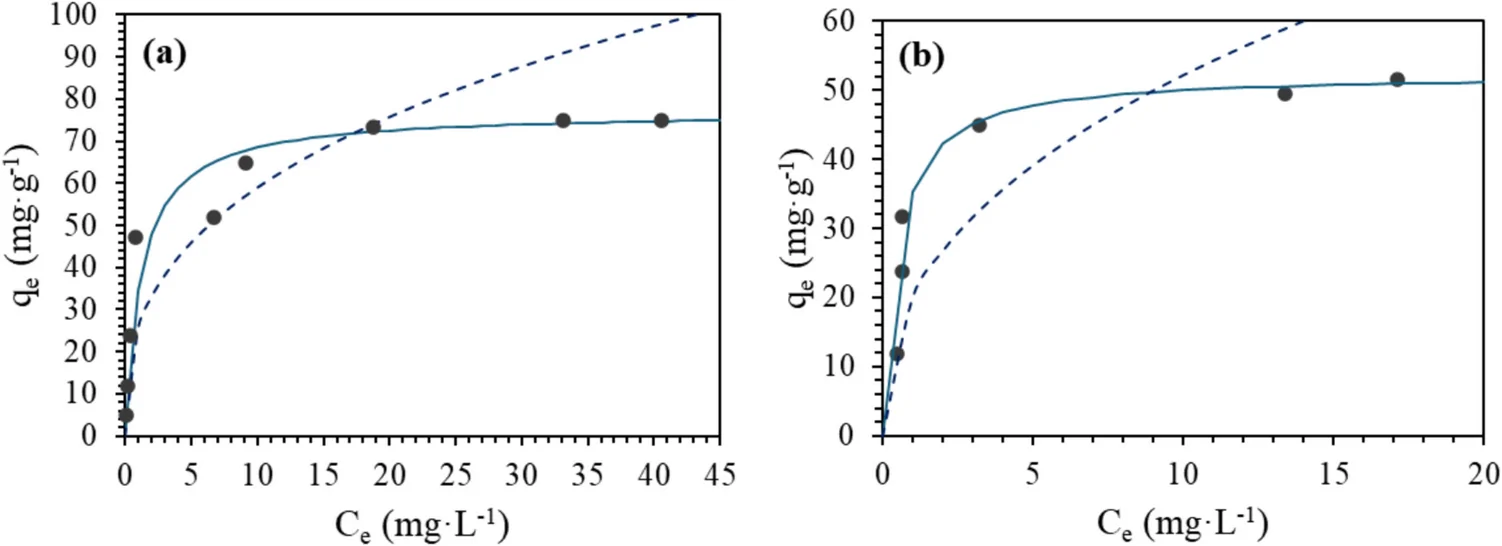
Adsorption isotherms of MB at 25 °C on **a** AC-650–2 and **b** MAC-3. Continuous lines correspond with Langmuir, and dashed ones with Freundlich fittings

Figure 4 a and b show the graphical representation of *q*_*e*_ vs *C*_*e*_ for AC-650–2 and MAC-3 at 25 °C. Similarly, the parameters obtained from each model are presented in Table 6.

**Table 6:** Parameters obtained from the adsorption isotherms of AC-650–2 and MAC-3 at 25 °C. *K*_F_ is in (mg·g^−1^) (L·mg^−1^)^1^/*ⁿ*, *q*_mon_ in mg·g^−1^, and *K*_L_ in L·mg^−1^

	Freundlich				Langmuir			
	*K*_F_	1/*n*	*n*	*R*^2^	*q*_mon_	*K*_L_	*R*^2^	*R*_L_
**AC-650–2**	25.621	0.360	2.774	0.885	76.336	1.139	0.999	0.017
**MAC-3**	24.746	0.294	3.398	0.697	53.763	1.069	0.997	0.018

The Freundlich model presented 1/*n* values between 0 and 1, indicating that adsorption is favorable. However, there was a better fit of the data with the Langmuir model due to the higher regression coefficient, *R*^2^, in both cases. Therefore, since the Langmuir model provided the best correlation, it can be concluded that the adsorption of MB on these ACs follows a monolayer adsorption process without interactions between the adsorbed molecules. This is consistent with that reported by other authors that synthesized SS-based ACs and used MB as probe molecule (Lu et al. 2023). The value of *R*_L_ was between 0 and 1 (see Table 6), which confirms that the adsorption process is favorable.

The maximum adsorption capacity, *q*_mon_, for AC-650–2 was 76.34 mg·g^−1^. This result is higher than that reported by other authors that synthesized SS-based ACs using other acid precursors. For example, Martin et al*.* reported MB adsorption of 14 mg·g^−1^ for a SS-based AC fabricated using H_2_SO_4_ (Martin et al. 2002). Rozada et al. reported *q*_mon_ values of 24.5 mg·g^−1^ and 102 mg·g^−1^ for the adsorption of MB onto SS-based ACs prepared with sulfuric acid and ZnCl_2_, respectively (Rozada et al. 2005). Nunthaprechachan et al. reported that the MB adsorption was between 27.4 and 29.3 mg·g^−1^ for SS-based ACs prepared using ZnCl_2_, KOH, or HNO_3_ as activating agents (Nunthaprechachan et al. 2013).

Higher MB adsorption capacity was reported for AC prepared from SS using K-containing activating agents. For instance, Lu et al. prepared SS-based ACs using KHCO_3_ and KOH in the chemical activation stage and found that the AC prepared with KHCO_3_ presented a higher mesopore volume, and thus, better adsorption of MB. In that work, MB adsorption increased with increasing carbonization temperatures, up to 800 °C, and the maximum adsorption of MB was 162 mg·g^−1^ (Lu et al. 2023). The highest MB adsorption reported for a SS-based AC was 588.2 mg·g^−1^ (at pH > 8), for an AC synthesized in two steps; a hydrothermal treatment followed by chemical activation with KOH. It must be noted that adsorption at pH 6.1 was about 250 mg·g^−1^ for the same material (Khoshbouy et al. 2019). The isotherm study in this work was done at the natural pH of the MB solution.

Despite the evidence of better adsorption results for materials synthesized using non-acid activating agents, as mentioned in the introduction, in this work, H_3_PO_4_ was chosen as activator to simplify the fabrication procedure, and to avoid the need of providing an inert atmosphere in the carbonization stage.

Regarding MAC-3, this material exhibited an adsorption capacity of MB of 53.76 mg·g^−1^. No other reference was found in which MAC was synthesized from a SS-based AC. However, a MB adsorption capacity of 47.44 mg·g^−1^, slightly lower than that reported in this work, was determined for a SS-based magnetic biochar, prepared by pyrolysis (Zeng et al. 2021).

#### Adsorption kinetics

Kinetic studies were carried out at different pH values, namely 4 and 8, and using different water matrices, distilled and tap water, for 15 and 50 mg·L^−1^ MB solutions, using MAC-3. For comparison purposes, the kinetic study was also done for AC-650–2 at pH 4 using a 15 mg·L^−1^ MB solution prepared with distilled water. These experiments allow to determine the effect of pH, adsorbate concentration, and water matrices in the adsorption process.

**Fig. 5:**
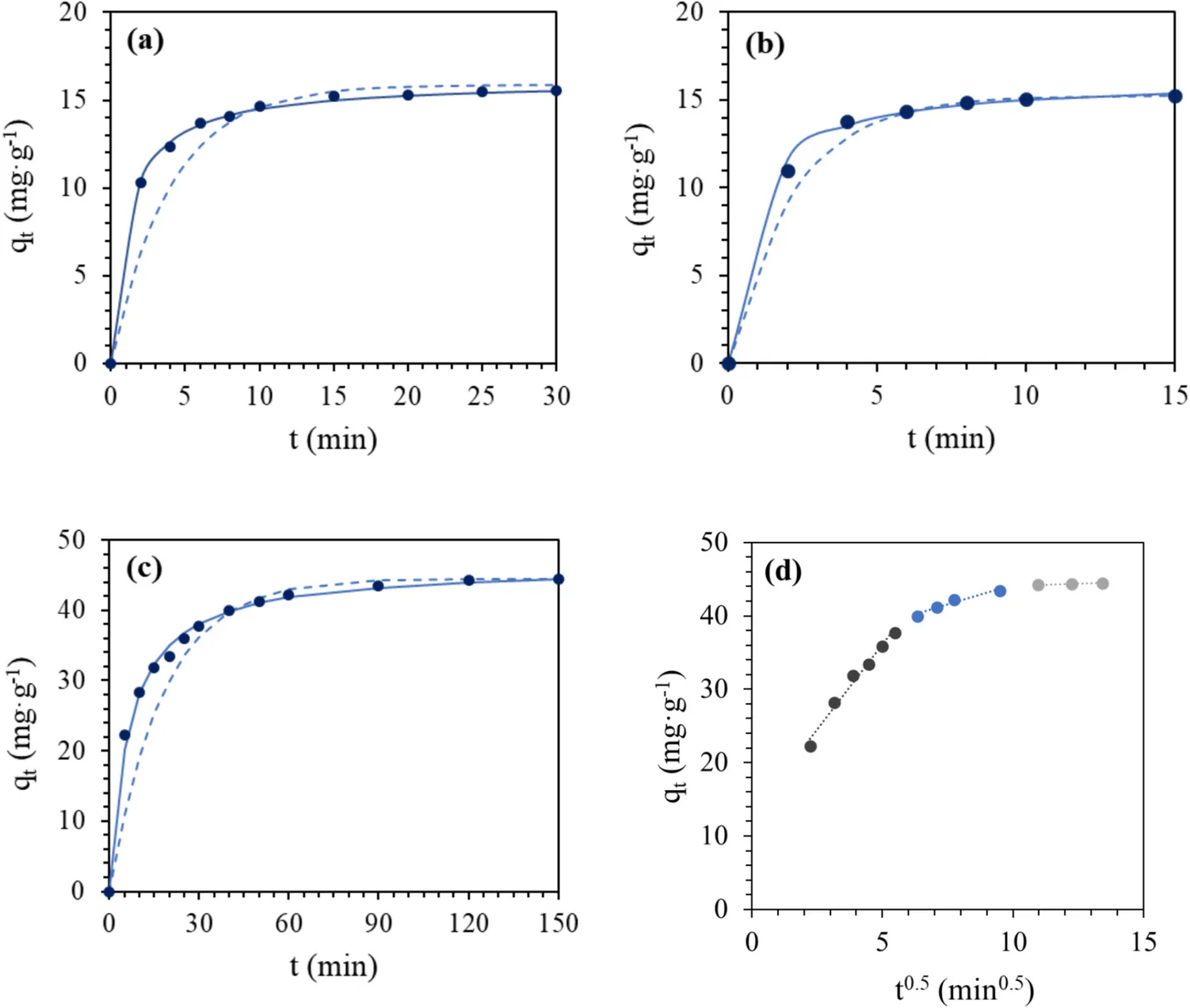
Adsorption kinetics for MAC-3 in tap water at 25 °C. **a** 15 mg·L^−1^ MB at pH 4. **b** 15 mg·L^−1^ MB at pH 8. **c** 50 mg·L^−1^ MB at pH 8. Continuous lines correspond with PSO, and dashed ones with PFO fittings. Intraparticle diffusion model fitting: **d** 50 mg·L.^−1^ MB at pH 8

Results are shown in Fig. 5 and in Table 7. It was observed that the PSO model fits better the experimental data than the PFO model. The calculated *R*^2^ was greater than 0.99 in all experiments. This model assumes that the rate-limiting step is chemisorption, where the adsorption rate of MB depends on the adsorbent’s capacity and not on the concentration of the adsorbate (Lu et al. 2023). Two stages can be differentiated in all cases. The first stage is a rapid adsorption of MB due to the abundance of empty sites, followed by a second stage where the available sites become saturated. Therefore, more time is required in the second stage to reach equilibrium (Sanz-Santos et al. 2022).

**Table 7:** Kinetic parameters obtained from MAC-3, for the adsorption of different concentrations of MB, using distilled and tap water at pH 4 or 8 and T = 25 °C. Kinetic parameters obtained from AC-650–2 for the adsorption of 15 mg·L^−1^ MB at pH 4 in distilled water. The concentration of MB in mg·L⁻^1^, *q*_*e*_ in mg·g⁻^1^, *k*_1_ in min⁻^1^, and *k*_2_ in g·mg⁻^1^·min⁻^1^

	MB (mg·L^−1^)	pH	Water matrix	*q*_*e*, exp_ (mg·g⁻^1^)	PSO		
					*q*_*e*, calc_ (mg·g⁻^1^)	*k*_2_ (g·mg⁻^1^·min⁻^1^)	*R*^2^
**MAC-3**	15	4	Distilled	15.85	17.01	0.0495	0.9992
	15	8	Distilled	16.16	16.56	0.1713	1.0000
	15	8	Tap	15.26	16.16	0.0792	0.9995
	50	8	Distilled	45.61	46.08	0.0017	0.9914
	50	8	Tap	44.49	46.30	0.0033	0.9998
**AC-650–2**	15	4	Distilled	16.86	16.86	8.7912	1.0000

The rate constant was 177 times higher for the AC-650–2 than for the magnetic AC (MAC-3), for the adsorption of 15 mg·L^−1^ MB at pH 4 in distilled water (see Table 7). Even though, both materials adsorbed, once adsorption equilibrium was reached, 100% of the MB concentration present in the media.

No similar studies were found regarding the use of MB as an adsorbate to test MACs synthesized from SS. Hence, the synthesis of MAC activated with ZnCl₂ from coconut shells was used for comparison. In that study, with an initial concentration of 100 mg·L^−1^ of MB, at 25 °C and natural pH, an experimental *q*_*e*_ of 20.8 mg·g⁻^1^ was achieved (Yağmur and Kaya 2021). In comparison, in this study, higher *q*_*e*_ values were obtained for the experiments done with 50 mg·L^−1^ MB at pH 8.

The effect of MB concentration on the adsorption kinetics was studied by conducting experiments at pH 8 with both distilled and tap water. Namely, 15 and 50 mg·L^−1^ MB were used. In all cases, over 90% of the MB was adsorbed by the MAC once the adsorption equilibrium was reached. The rate constant was 100 times higher when the MB concentration was lower, which meant that the adsorption equilibrium was reached faster in that case. Regarding the water matrix, the rate constant was 2.16 times higher when distilled water was used, for the experiment with 15 mg·L^−1^ MB. This was expected, because the ions present in the tap water could interfere with the adsorption of MB onto the MAC (Quesada et al. 2022). For the higher MB concentration, 50 mg·L^−1^, the rate constant was almost equal for the experiments done in distilled water and tap water. Thus, the effect of the ions present in tap water did not affect the adsorption process at higher adsorbate concentrations.

The adsorption mechanism was investigated using the Weber and Morris intraparticle diffusion model (see Fig. 5d), which has been studied by other authors for the adsorption of MB onto different adsorbents. They found that the adsorption of MB follows multiple stages (Gouamid et al. 2013, Kurnia et al., 2023). In this study, the same findings were observed. Thus, from Fig. 5d was observed that the plot of *q*_*t*_ vs *t*^0.5^ presents three stages. This indicates that three different processes are responsible for the adsorption of MB onto the material. The first slope, which is the higher slope, represents the external surface or instantaneous adsorption; the second slope represents the stage of intraparticle diffusion, where the adsorbate diffuses and is adsorbed into the pores; and the third one, which has the lowest slope, is due to the adsorption equilibrium (Gouamid et al. 2013). The intraparticle diffusion rate constant was depicted from the second slope, and was 1.0657 mg·g^−1^·min^−0.5^.

**Fig. 6:**
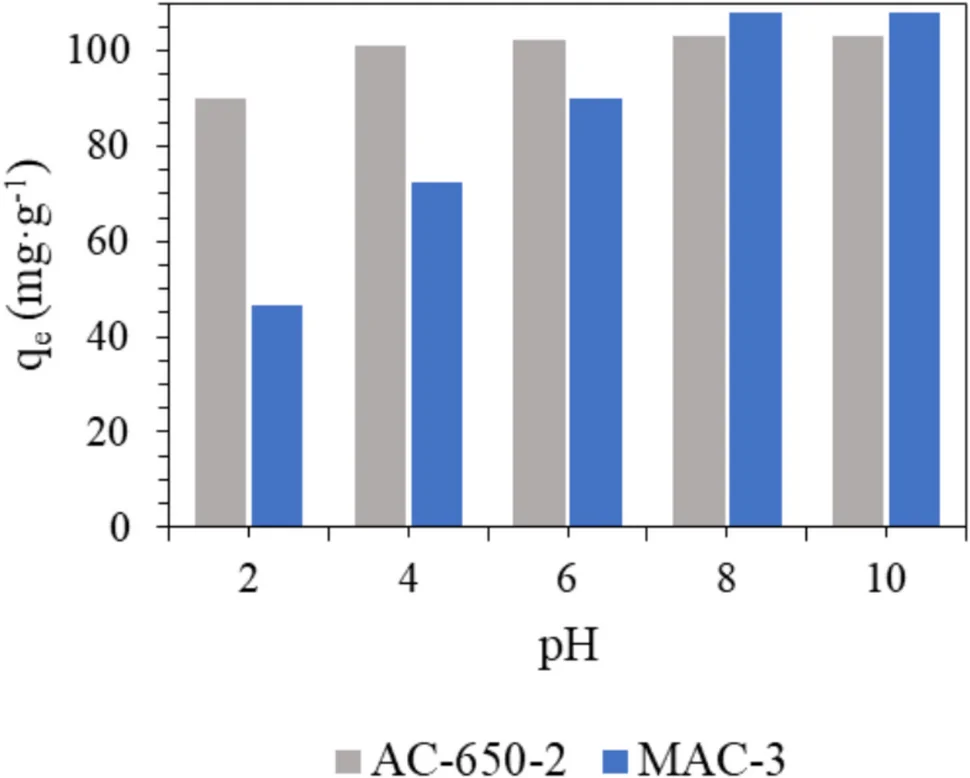
Adsorption capacity, *q*_*e*_, of AC-650–2 and MAC-3 for the adsorption of 100 mg·L^−1^ MB at different pH at *T* = 25 °C

Regarding pH, results revealed that the adsorption of MB was favored at basic pH. Such, the adsorption rate constant was 3.46 times higher at pH 8 than at pH 4, in distilled water. Similarly, it was 1.42 times higher at the basic pH when tap water was employed. The effect of pH on the adsorption capacity can be depicted in Fig. 6.

The higher adsorption capacity found at the basic pH can be explained following the pH_pzc_ of the MAC and the pK_a_ of MB.

On the one hand, the pH_pzc_ of MAC-3, that is, the pH at which the activated carbon has zero net electrical charge, was 4.7. The pH_pzc_ of AC-650–2 is 3.0, as shown in Table 2. Thus, the surface of the material will be positively charged at pH < pH_pzc_ and negatively charged at pH > pH_pzc_.

On the other, MB (pK_a_ = 3.8) is present in aqueous solution in its cationic form (MB^+^) at pH > 3.8, and, specifically, it has been reported to be present as MB^+^ at pH > 6 (Salazar-Rabago et al. 2017). For this reason, at basic pH, both AC-650–2 and MAC were negatively charged, and MB was in its cationic form, and, thus, adsorption was favored. The highest adsorption was achieved at a lower pH for AC-650–2 compared to MAC-3 due to its lower pH_pzc_.

#### Adsorption thermodynamics

The calculated thermodynamic parameters are shown in Table 8. The negative Δ*G* values indicate that the adsorption of MB occurs spontaneously on the adsorbents studied in this work. Additionally, lower Δ*G* were observed at higher temperatures, which concludes that adsorption is favored at higher temperatures. This agrees with the findings of other authors for the adsorption of MB onto activated carbon (Jawad et al. 2016).

**Table 8:** Thermodynamic parameters of the adsorption of MB onto AC-650–2 and MAC-3

	Δ*H* (J·mol^−1^)	Δ*S* (J·mol^−1^·K^−1^)	Δ*G* (J·mol^−1^)		
			283 K	298 K	313 K
**AC-650–2**	1714.1	21.5	− 4362.0	− 4716.3	− 5007.3
**MAC-3**	13,708.0	53.1	− 1506.1	− 1716.2	− 3098.2

The positive Δ*H* describes an endothermic adsorption process, and the positive Δ*S* value suggests a higher randomness at adsorbate-solution interface during adsorption, as described by other authors for the adsorption of MB onto activated carbon (Theydan & Ahmed 2012).

### Reuse and regeneration studies

Regeneration studies were carried out with the aim of recovering the saturated material, since from an environmental and economic point of view it is fundamental. For this purpose, the AC and MAC were first saturated with a 50 mg·L^−1^ MB solution in tap water. Adsorption experiments were performed at pH 8, according to the best conditions for adsorption depicted above.

In literature, AC regeneration can be achieved by different methods, as indicated above. The ones studied in this work were chemical generation (for AC-650–2 and MAC-3) and advanced oxidation (for MAC-3). The reason for this is the simplicity of the process in comparison to others. In this sense, both regeneration methods can be applied in situ, and no additional equipment is needed for the regeneration process.

In the chemical regeneration process, several characteristics must be considered, such as the solubility of the adsorbate in the solution used to regenerate the AC, or the pH at which desorption can be favored for the specific adsorbate and AC studied. Thus, the regeneration technique must be studied individually for each specific purpose.

Regarding the use of different ACs, from different nature, to adsorb MB, some authors have reported the regeneration of ACs saturated with MB via acid washing of the material. Naushad et al*.* studied the regeneration of AC using three different acids, namely 0.1 M solutions of HCl, H_2_SO_4_, or HNO_3_, and found that, among them, HCl offered best recovery results (Naushad et al. 2019). For this reason, a 0.1 M solution of HCl (pH ≈ 1.7) was chosen as one of the regeneration solutions in this study.

Despite this, Li et al. studied the regeneration of AC, also saturated with MB, using 1 M HCl or NaOH solutions, or a 90% ethanol solution, and found better recycle results when the AC was washed with ethanol, followed by NaOH, and lastly HCl (Li et al. 2020). Other authors used 0.1 M NaOH solution for the same purpose and achieved almost 65% recovery of the AC after 5 cycles (Somsesta et al. 2020). Following this, 0.1 M NaOH and 70% ethanol solutions were also studied in this work as regeneration solutions.

Lastly, the lixiviation of the iron contained in the MAC-3 was studied at different pH values to determine if a Fenton reaction could be studied as regeneration process for the adsorbent. For this, MAC-3 was left in deionized water at pH 2, 3, or 4 (adjusted with HCl) for 1 h and, next the concentration of iron in the supernatant was determined. The lixiviation of iron was 1.44 mg·L^−1^ at pH 2, and less than 0.30 mg·L^−1^ at pH 3 or 4. If the impregnation time was increased to 24 h, the lixiviation of iron at pH 2 increased to 3.60 mg·L^−1^.

An additional regeneration experiment was done with MAC-3, at pH 2, adjusted with HCl, adding different concentrations of H_2_O_2_ to the system. In the Fenton reaction, the concentration of both iron and H_2_O_2_ must be optimized. Theoretically, 2.125 g H_2_O_2_ g^−1^ COD (chemical oxygen demand) are needed to completely oxidize a contaminant (Genli et al. 2021). Thus, for the 50 mg·L^−1^ MB used in this study, about 150 mg·L^−1^ H_2_O_2_ would be required.

However, it has been proved that an excess H_2_O_2_ acts as •OH scavenger, and thus, reduces the reaction rate of the Fenton process (Melián et al. 2023). The H_2_O_2_ that the system really needs will depend on two additional factors: the amount of MB that can be efficiently desorbed from the AC, and, most importantly, the concentration of iron that will be available for the Fenton reaction. Suppose that this concentration will be the iron lixiviated from the MAC-3, that is, 3.60 mg·L^−1^ in 24 h. The optimal H_2_O_2_:Fe^2+^ ratio for the Fenton reaction of MB has been reported to be 12 mmol, although the optimal ratio depends on the specific case study (Dutta et al. 2001). Therefore, in such case, the concentration of H_2_O_2_ in the present work should be 27 mg·L^−1^. Considering all the above, in this study, three concentrations of H_2_O_2_ were studied for the regeneration of MAC-3: 30, 100, and 200 mg·L^−1^.

**Fig. 7:**
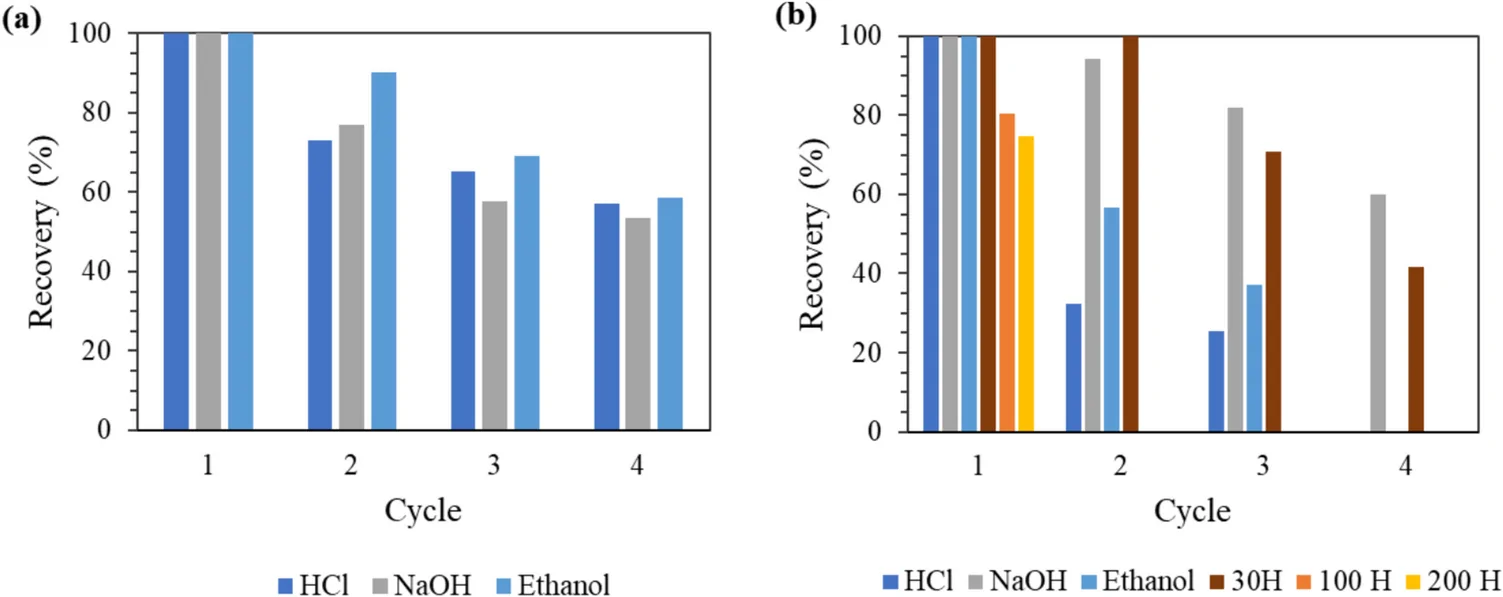
Recovery of the adsorption capacity, *q*_*e*_, of AC-650–2 (**a**) and MAC-3 (**b**) for four cycles. The regeneration solutions were HCl, NaOH, ethanol, or H_2_O_2_ at pH 2 (adjusted with HCl), 30H (30 mg·L^−1^ H_2_O_2_), 100H (100 mg·L^−1^ H_2_O_2_), and 200H (200 mg·L.^−1^ H_2_O_2_)

Regeneration results are shown in Fig. 7. Regeneration efficiency results for AC-650–2 are in line with those reported by Naushad et al. (Naushad et al. 2019), despite the different nature of the AC precursor used in that study and in the present one. On the fourth cycle, the recovery was about 60%, similar for the three regeneration methods (HCl, NaOH, and ethanol).

Regarding MAC-3, the recovery differs considerably for the different regeneration solutions. Recoveries higher than 50% in the fourth cycle were only achieved when NaOH or 30 mg·L^−1^ H_2_O_2_ were used. The best regeneration solution was NaOH, with 60% recovery in the fourth cycle. Results are similar to those reported by Do et al., who studied the regeneration of a MAC synthesized from a commercial AC to adsorb methylene orange (Do et al. 2011).

It must be noted that the addition of the higher concentrations of H_2_O_2_, 200 and 100 mg·L^−1^, significantly reduced the adsorption capacity of MAC-3 in the first regeneration cycle compared to the other regeneration alternatives. This could be attributed to an excess H_2_O_2_, that can interfere with the surface chemistry of the material, reducing its adsorption capacity.

For comparison purposes, an additional experiment was done to regenerate AC-650–2 with 30 mg·L^−1^ H_2_O_2_ at pH 2 (results not shown). In that case, the recovery decreased drastically in the first regeneration cycle. Thus, the presence of iron (from MAC-3) in the media was fundamental for the recovery of the material with H_2_O_2_, which suggests that, effectively, the Fenton reaction was responsible for the regeneration in the MAC-3.

### Economic study

The energy requirements along the synthesis process of AC-650–2 and MAC-3, as well as the cost derived from the required reagents, is shown in Table 8. The reagent cost per unit (kg or L) was given by the suppliers. The washing process of the synthesized AC was excluded from calculation, because the laboratory washing system was not efficiently insulated and much heat loss was produced during the washing time (36 h). The cost of electricity was 0.1217 €·kWh^−1^, taken from European official statistics for Spain (first semester 2024) (Eurostat 2024). For MAC-3, only the cost of reagents was taken into consideration.

To our knowledge, only one recent study has reported the cost of producing SS-based AC. In that study, the authors used KOH as activating agent and carbonized the material under nitrogen at 800 °C. The cost they estimated, including energy and reagent costs, was € 17.53 per kg AC if the material was synthesized from wet SS, and 8 € per kg if the humidity of the SS was first reduced to 80% (Martínez-Alvarenga et al. 2024). Martinez-Alvarenga et al. reported that the energy consumption was 2404 MJ·t^−1^ wet SS during the drying stage, and 6353 MJ·t^−1^ wet SS during pyrolysis.

The cost calculated in the present work for AC-650–2 was 5.41 €·kg^−1^ AC, which is slightly lower than that reported by the mentioned authors, considering that, in this work, the SS used for the fabrication of the AC was in dry basis. The lower cost is mainly due to lower carbonization temperature used in this work, and to the absence of nitrogen requirement.

The production of MAC-3 is not cost effective, as shown in Table 9. Moreover, the adsorption capacity of that material is lower, as mentioned above, and the recovery capacity is similar to that of AC-650–2.

**Table 9:** Energy requirements and cost of production of AC-650–2 and MAC-3

	AC-650–2	MAC-3
Energy requirements		
Drying (kWh·kg^−1^ AC)	2.16	-
Carbonization (kWh·kg^−1^ AC)	17.5	-
Energy costs		
Drying (€·kg^−1^ AC)	0.26	
Carbonization (€·kg^−1^ AC)	2.13	
Reagent costs		
H_3_PO_4_ 85% (€·kg^−1^ AC)	3.02	-
FeCl_3_ (€·kg^−1^ AC)	-	6.97
FeCl_2_·4H_2_O (€·kg^−1^ AC)	-	11.40
HCl 2 M (€·kg^−1^ AC)	-	3.15
NH_3_ 25% (€·kg^−1^ AC)	-	8.98
AC-650–2 (€·kg^−1^ AC)	-	1.81
Total cost (€·kg^−1^ AC)	**5.41**	**32.31**

## Conclusions

In this work, dry SS was used to synthesize AC. The adsorbents were fabricated using a facile method, through chemical activation with phosphoric acid, and carbonized under a self-generated atmosphere, to avoid the need of using an inert atmosphere and, thus, the cost associated to this. Seven ACs were fabricated, to study the effect of carbonization temperature and impregnation ratio of the activating agent, on the resulting material. The efficiency of the materials was first determined experimentally with the MB number. The AC that exhibited the highest adsorption capacity was the one fabricated with 2 g H_3_PO_4_·g^−1^ precursor and carbonized at 650 °C (AC-650–2), with a MB number of 140. The MB number of a commercial AC used for comparison purposes was 160. The iodine number of the optimized AC was in line with that reported for commercial ACs.

The adsorption isotherms revealed that the data fitted well with the Langmuir model, and the maximum adsorption capacity for AC-650–2 was 76.34 mg·g^−1^, at 25 °C. This result was higher than that reported by other authors that synthesized SS-based ACs using other acid precursors. The adsorption kinetics followed the PSO model. Adsorption was favored at basic pH, which is consistent with the pK_a_ of the adsorbate (methylene blue) and the pH_PZC_ of the adsorbent. Regarding the water matrix, adsorption was slightly inhibited when tap water was used instead of distilled water.

A total of four magnetic ACs (MACs) were synthesized using different AC-650–2 loads. The magnetic materials were fabricated using the Massart’s method, with iron salts. The MB number of the optimized MAC, that was MAC-3, the one synthesized with the highest AC load (3 g), presented a maximum adsorption capacity of 56.76 mg·g^−1^, at 25 °C.

The recovery of the saturated materials was studied using chemical washing, namely with HCl, ethanol or NaOH. Additionally, MAC-3 was also recovered, at pH 2 (adjusted with HCl) with H_2_O_2_. This last regeneration method was based on the lixiviation of iron from MAC-3 (3.60 mg·L^−1^ at pH 2 in 24 h), that could be used to produce a Fenton reaction with H_2_O_2_, and, thus, mineralize the desorbed methylene blue. The most effective recovery methods were washing with NaOH and the Fenton reaction, using 30 mg·L^−1^ H_2_O_2_.

The cost of producing the materials was 5.41 €·kg^−1^ for AC-650–2, and 32.31 €·kg^−1^ for MAC-3. The cost of AC-650–2 is slightly lower than that reported by other authors for the synthesis of SS-based AC.

Future investigations should be aimed at the study of efficient and cost-effective regeneration techniques for AC, that combine both the recovery of the adsorption capacity of the material and the mineralization of the contaminants desorbed during regeneration. In this sense, life-cycle assessment could provide reliable information on the most appropriate synthesis and regeneration procedures for SS-based AC.

## Data Availability

The datasets used and/or analyzed during the current study are available from the corresponding author upon reasonable request.
